# Prevalence of *Calodium hepaticum* and *Cysticercus fasciolaris* in Urban Rats and Their Histopathological Reaction in the Livers

**DOI:** 10.1155/2014/172829

**Published:** 2014-11-05

**Authors:** Bharathalingam Sinniah, Muniandy Narasiman, Saequa Habib, Ong Gaik Bei

**Affiliations:** Laboratory Based Medicine, Faculty of Medicine, Universiti Kuala Lumpur Royal College of Medicine Perak, No. 3 Jalan Greentown, 30450 Ipoh, Perak, Malaysia

## Abstract

Humans can get infected with several zoonotic diseases from being in close contact with rats. This study was aimed at determining the prevalence and histopathological changes caused by *Calodium hepaticum* and *Cysticercus fasciolaris* in infected livers of wild caught urban rats. Of the 98 urban rats (*Rattus rattus diardii* and *Rattus norvegicus*) autopsied, 64.3% were infected; 44.9% were infected with *Caladium hepatica*, 39.3% were infected with *Cysticercus fasciolaris*, and 20.4% were infected with both parasites. High infection rates suggest that urban rats are common reservoir for both parasites, which are potentially a threat to man. *Calodium hepaticum* infections were identified by the presence of ova or adults in the liver parenchyma. They appear as yellowish white nodules, measuring 1–7 mm in diameter or in streaks scattered widely over the serosal surface of the liver. *Cysticercus fasciolaris* infections are recognized morphologically by their shape (round or oval) and are creamy white in colour. Histological studies of *Calodium hepaticum* showed areas of granulomatous lesions with necrotic areas around the dead ova and adults. In almost all cases, the rats appeared robust, looked healthy, and showed no visible signs of hepatic failure despite the fact that more than 64.0% of their livers were infected by either one or both parasites.

## 1. Introduction

Rats are the most common and widespread of all mammals in the world, making it difficult to estimate their numbers, which probably run into the billions. They are responsible for the transmission of several zoonotic diseases to man, which include parasites and microbes. Man gets infected with many rodent pathogens primarily through bites, scratches, and the consumption of food or drinks contaminated with rodent droppings or urine. Besides transmitting diseases, they eat almost anything and destroy properties. Currently, most large cities and towns in developing and developed countries face a growing menace from the rat population. Rats are definitive host for* Calodium hepaticum* and intermediate host for* Cysticercus fasciolaris*. The nematode parasite* Calodium hepaticum* (syn.* Capillaria hepatica*, Bancroft, 1983) has a global distribution and is commonly reported in rodents [1, 2] and to a lesser extent in dogs [3, 4], cats [5], primates [6], and humans [5, 7–9].* Calodium hepaticum* is a common parasite which infects rodents worldwide but rarely in humans. Currently 163 human cases of* Calodium hepaticum* (72 reports of hepatic capillariasis, 13 serologically confirmed infections, and 78 observations of spurious infections) have been reported from different parts of the world [9, 10].* Calodium hepaticum* ova are deposited by female worms in clusters in the parenchyma tissue of the liver and are only released into the environment following the death of its host due to natural causes, predation cannibalism, or decay. Consumption of infected livers is the main cause of spurious infection, where the eggs are released in the faeces without causing disease. Human infection with* Taenia taeniaeformis* is rare and only few cases have been reported [11]. Hepatic calodiasis is mainly diagnosed by liver biopsy or necropsy.* Cysticercus fasciolaris* is the larval stage of the cestode,* Taenia taeniaeformis* (Batsch, 1786) (syn.* Hydatigera taeniaeformis*) also known as cat tapeworm. The pathogenesis of these two parasites is distinct.* Calodium hepaticum* infection causes multifocal granulomatous inflammation and is directly associated with the presence of live, dying, or dead worms or their eggs. Septal fibrosis is commonly seen in animals infected with* C. hepaticum* [12–14].* Cysticercus fasciolaris* infections induce vigorous fibroplasia and progressive inflammation within the liver parenchyma [12, 13]. The aim of this study was to determine the prevalence of* Cysticercus fasciolaris* and* Calodium hepaticum* coinfection and their pathology in rodent livers to help better understand the infection in humans.

## 2. Materials and Methods

A total of 104 rodents, comprising 52* Rattus norvegicus*, 46* Rattus rattus diardii*, and 6* Mus musculus,* were trapped from the urban areas of Ipoh, Malaysia. Ipoh was chosen as the area of study as there was a campaign by the local municipal council to control the rat population because rats were becoming a big public health problem. The rodents were trapped in the vicinity of wet markets and drains behind restaurants and food courts. Rats were captured using wire traps that measured 29 × 22 × 50 cm. For bait, we used either dried salted fish, or slices of bread spread with peanut butter or cheese. Data of each rodent necropsied were recorded, including locality, date of capture, and gender. Fifty-one female and forty-seven males were trapped at night and transported to the laboratory in the morning. The rats were necropsied and examined within 24 hours of capture. Animal procedures were conducted in adherence to the Ethical Committee of the University. Each trap with the live rat inside was individually placed into a big bucket with a lid. The animals were anaesthetized by placing cotton wool soaked with chloroform into the bucket and covered for 2-3 minutes. The anaesthetized rat was removed from the cage and sacrificed by severing the aorta. Gross examination of the liver was initially conducted to screen for* Calodium hepaticum* and* Cysticercus fasciolaris* infections. A small portion of the infected liver was removed from each rat, fixed in 10.0% neutral-buffered formal-saline (pH 6.9), and processed for paraffin embedding. The tissues were sectioned at 5 *μ*m in thickness. In addition, all macroscopic abnormalities were also examined. The cut sections were stained with haematoxylin and eosin (H&E) and examined under light microscopy for histopathological reactions. Infections were confirmed by demonstrating the presence of ova or adults of* C. hepaticum* in the liver and* Cysticercus fasciolaris* was confirmed by breaking the cyst and exposing the larva.

## 3. Results

**Figure 1:**
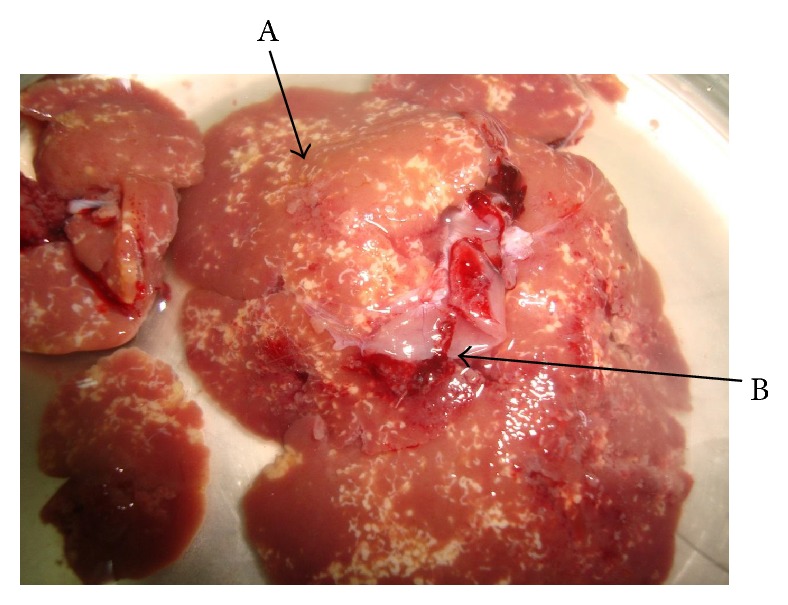
Liver of urban wild rodent showing coinfection with* Calodium hepaticum* and* Cysticercus fasciolaris*.* Calodium hepaticum* (A) appears as yellowish white patches/tracts or streaks on the liver surface. The cysts of* Cysticercus fasciolaris* (B) are embedded in the liver (arrow) containing the larva.

**Figure 2:**
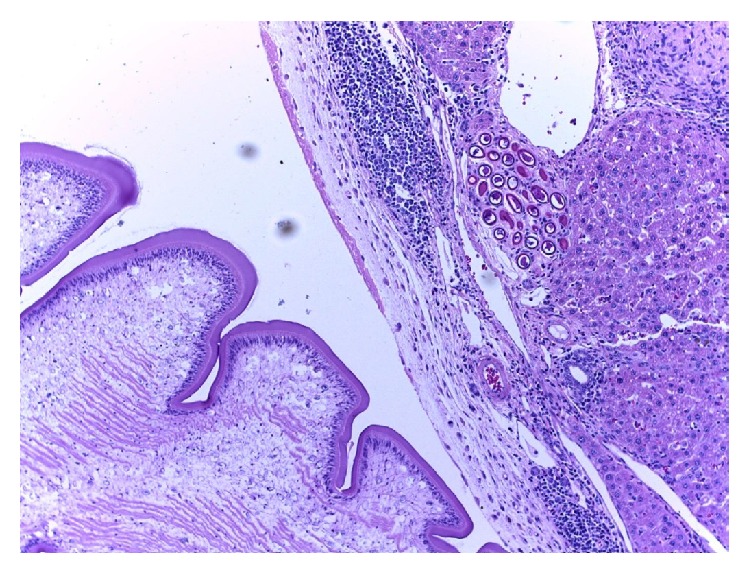
*Cysticercus fasciolaris* is within a cyst which is surrounded by connective tissues and inflammatory cells. Eggs of* Calodium hepaticum* are shown in a cluster within the parenchyma tissues (haematoxylin and eosin stain: ×10).

**Figure 3:**
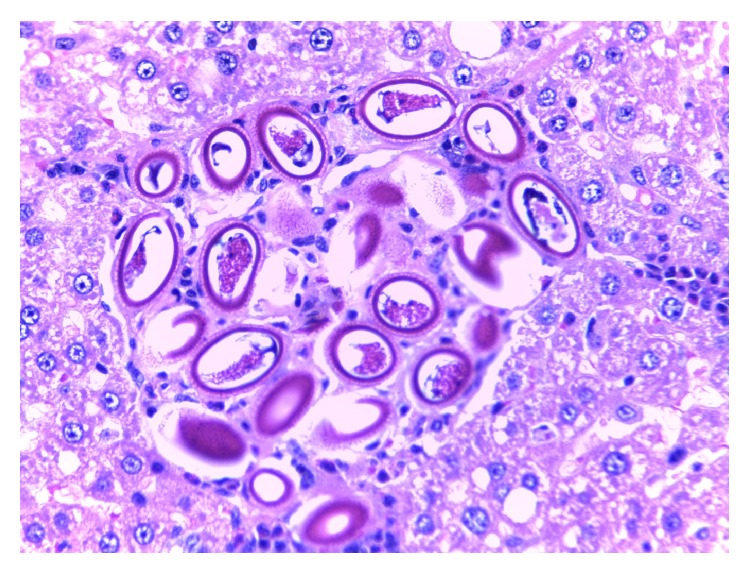
Cluster of* Calodium hepaticum* eggs surrounded by granulomatous lesion. Bioperculated ova with polar prominence at each end that are characteristic of* C. hepaticum* are seen (haematoxylin and eosin stain: ×40).

**Figure 4:**
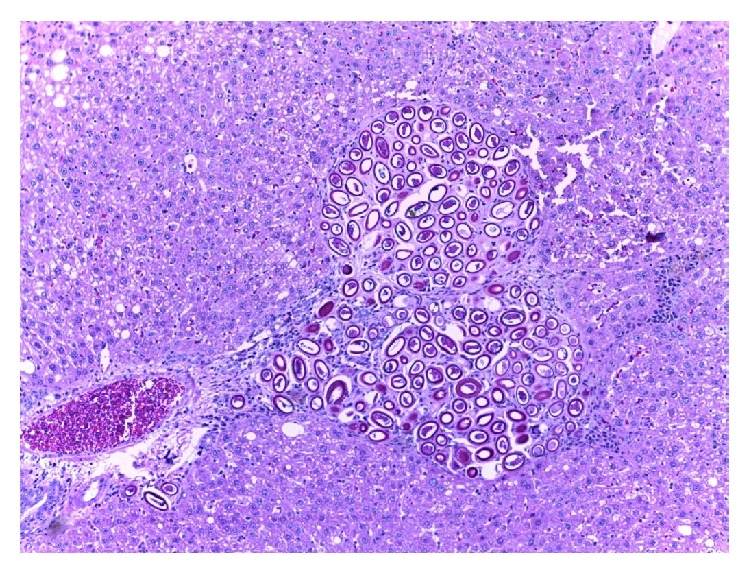
Clusters of* C. hepaticum* eggs surrounded by granulomatous lesions (H&E stain ×10).

**Figure 5:**
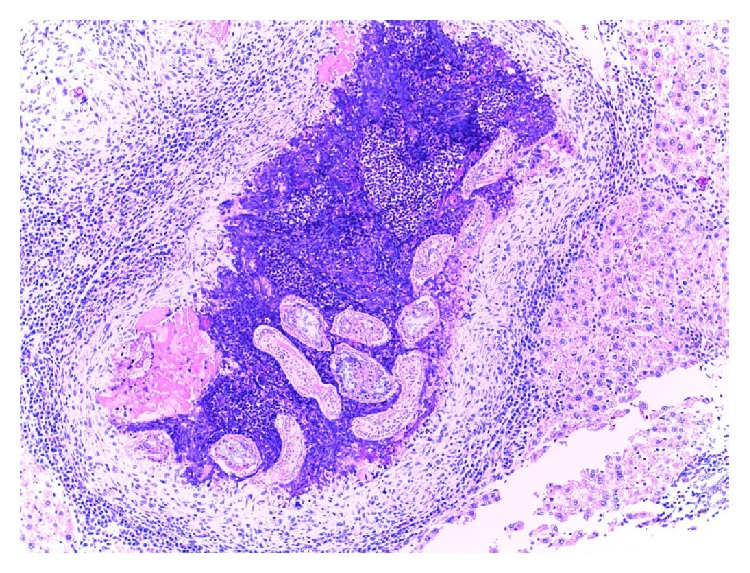
Dead worms and eggs are surrounded by an acute inflammatory reaction containing a central area of necrosis, calcification, and fibrosis (H&E stain: ×10).

Of the 98 rats examined, 64.0% were infected, of which 44.9% were positive with* Calodium hepaticum*, 39.3% were positive with* Cysticercus fasciolaris*, and 20.4% were positive with both as shown in Table 1. None of the six shrews examined were infected with these two parasites. Gross examination of the infected livers showed firm whitish yellow nodules (1–7 mm) or appeared in patches or as irregular streaks, randomly scattered in the serosal surface.* Cysticercus fasciolaris cysts* measured, on an average, 9–14 mm in diameter. Each cyst contained a single larva measuring 14–31 cm in length, with a scolex containing two rows of hooks, 4 suckers followed by a very long neck. The infected rats mostly had multiple cysts, which appeared creamy white in colour, oval or round in shape, embedded within the liver as shown in Figure 1. Prior to necropsy, none of the rats exhibited any adverse clinical signs. Histopathological studies showed that encapsulation by* Cysticercus fasciolaris* caused very little fibrotic changes in the rats. In some signs of inflammation with periportal eosinophil infiltrates, microabscesses and prominent Kupffer cells were seen. In some livers, the larvae were seen encapsulated by connective tissue surrounded by mild to moderate inflammatory infiltrates predominantly composing lymphocytes, macrophages, moderate eosinophils, and few large scattered fibroblasts. In few livers, mild fibrotic lesions were seen around the cyst, as shown in Figure 2.* Calodium hepaticum* ova are barrel-shaped and unembryonated and have typical bipolar plugs on either end with prominent radial striations on the outer layer of the egg shell. The eggs measured, on an average, 22 *μ*m in width and 51 *μ*m in length as shown in Figure 3. Histopathological section of infected livers showed areas of granulomatous reaction. These responses were more profound around the ova of* Calodium hepaticum* infection with fibrous inflammatory reaction, as shown in Figure 4. Tissue reaction around dead* Calodium hepaticum* worms or ova consisted of a large numbers of inflammatory cells, mainly mononuclear leukocytes, few polymorphs, and eosinophils, as shown in Figure 5. Microscopic studies showed multiple nodular microgranulomas and coalescing macrogranulomas with intralesional parasitic eggs. Occasionally adult worms were seen scattered in the liver. Small granulomas (microgranulomas) in the liver of some rats are comprised of solid aggregates of epithelioid macrophages surrounded by lymphocytes and eosinophils. In few cases, the granulomas contained no fragments of worms, eggs, or any trace of worm derived material. Thin and thick peripheral lesions are most commonly characterized by fibrous capsules, containing lymphocytes, small to moderate amounts of neutrophils, and plasma cells. The core of the nodules contained mainly neutrophils with necrotic debris, epithelioid cells, few multinucleated cells, eosinophils, and occasionally calcified materials. In addition the central hepatic vein was moderately dilated while the surrounding sinusoids in the livers contained erythrocytes and a few inflammatory cells. Fibrocellular septae, separated the hepatic parenchyma into irregular portions, were noted in the vicinity of granulomatous reaction. Periportal inflammatory infiltration and hepatocyte regeneration were also observed.

## 4. Discussion

*Calodium hepaticum* infection in rodents has been reported worldwide with prevalence rates ranging from 7.9% to more than 88.0% [1, 8, 10, 15]. In this study, 44.9% of rats were infected with* Calodium hepaticum* and 39.3% with* Cysticercus fasciolaris*. The adult female* Calodium hepaticum* deposits ova in clusters in the liver parenchyma and these eggs become encapsulated as a result of chronic inflammatory response of the host. The ova are lemon-shaped with thick egg shells and are striated with polar plugs at each end, measuring on average 22 *μ*m in width and 51 *μ*m in length. Macroscopically, infected livers showed diffuse, irregular yellowish white patches that appeared in streaks or small nodules on the external surface and within the liver, and in some cases adult worms and egg masses were found, in line with earlier reports [16–18]. Adult male worms die approximately forty days after infection, whereas females die within 60 days. Dead worms disintegrate and become “walled off” due to host response and gradually destroyed.

The clusters of ova which are trapped within the liver undergo fibrotic tissue responses [17, 19]. The worm material inside the fibrotic reaction may be viewed as a protective response. In the present study, we observed heavy lymphocytic infiltrates at the periphery of the egg containing granulomas. Our observations are consistent with studies conducted in other countries that showed similar granulomatous inflammation in the livers of naturally infected rats [8, 20]. This response is precipitated by dying and disintegrating adult worms and ova. The granuloma (macrogranuloma) formation is said to have an immunological basis and occurs as a consequence of an immune response mounted by the host, against egg-derived antigens [15, 21]. In support of such an idea, Solomon and Soulsby [22] reported finding circulating antibodies to egg antigens in infected mouse. Focal lesions with septal fibrosis are very often seen associated with* C. hepaticum* infections [17]. Septal fibrosis is commonly seen in more than 90.0% of infected livers and is represented by thin, straight fibrocellular/fibrous tissue septa that divide the liver parenchyma into hepatic nodules. Septal fibrosis extends from portal spaces and spreads toward neighbouring portal spaces and central veins, finally involving the entire organ, thus creating a septate mosaic pattern in the infected liver [17, 19, 23].

In rats,* Cysticercus fasciolaris* cysts can occur singly or in numerous numbers. The cyst wall is rough, and in chronic infections, it can cause irritation to the hepatic tissues, inducing slight local inflammatory reaction due to immune-related activities [24]. Microscopic observations showed mild inflammatory reactions around the cysts, infiltrated by lymphocytes, macrophages, eosinophils, and few scattered large fibroblasts. Rats infected with* Cysticercus fasciolaris* and other cestode larvae have the capacity to stimulate immune responses, which allow the larval stage to become invasive [25]. This irritation may stimulate and promote the hepatic cells around the cyst to develop and exhibit carcinogenic behaviour. The chemical reactions caused between the larvae and hepatic tissues may induce cellular changes which may develop into fibrosarcomas [26].

Histopathological observations in chronic infections showed plenty of fibroblasts with neoplastic characteristics similar to fibrosarcomas. [27, 28]. In chronic infection where the hepatic cysts are more than three months old, the larvae may induce fibrosarcomas in the liver tissue [24, 27, 28]. In man,* Calodium hepaticum* may cause the loss of liver cells, thereby resulting in the loss of liver function. Dead adult parasites can stimulate a rapid immune response in the host, leading to inflammation and encapsulation in collagen fibres leading to septal fibrosis and cirrhosis [15].

## 5. Conclusion

As of today there are no good policies on the control of rats for health and economic benefits. The laws for proper sanitation are not effectively implemented. As a result the prevalence of* Calodium hepaticum* and* Cysticercus fasciolaris* in rodents is high in many tropical countries and poses a health threat as large populations of humans live in close proximity to the rodent population. These rodents act as reservoir hosts for these parasites and are a potential risk for human infections in Malaysia as well as in other countries in the region. Cases of human infections reported from this region are from Thailand [29], India [30], Korea [31], and China [32–35]. In view of this and with the high prevalence of rats in this country, steps should be taken to prevent human infections. The effective solution lies in the hands of the communities. To achieve this there is a need to mobilize the communities to promote better health through self-help and group activities.

## Figures and Tables

**Table 1:** Prevalence of *Calodium hepaticum* and *Cysticercus fasciolaris* in liver of 98 urban rats.

Liver parasite	Number of positive	% positive
Single infection		
*Calodium hepaticum *	24/98	24.5
*Cysticercus fasciolaris *	19/98	19.4
Double infection		
*C. hepaticum* + *C. fasciolaris *	20/98	20.4
Total* C. hepaticum *infection	44/98	44.9
Total* C. fasciolaris *infection	39/98	39.3
Total liver infected	63/98	64.3
